# Severe Preeclampsia, Antiphospholipid Syndrome, and Ulnar Artery Thrombosis in a Teenage Pregnancy: A Rare Association

**DOI:** 10.1155/2018/1794723

**Published:** 2018-09-18

**Authors:** M. Patabendige, G. Barnasuriya, I. Mampitiya

**Affiliations:** ^1^University Unit of Obstetrics and Gynaecology, Teaching Hospital, Mahamodara, Galle, Sri Lanka; ^2^Teaching Hospital, Karapitiya, Galle, Sri Lanka; ^3^Department of Obstetrics and Gynaecology, Faculty of Medicine, University of Ruhuna, Galle, Sri Lanka

## Abstract

Antiphospholipid syndrome (APS) is associated with vascular thrombosis and pregnancy complications. It causes recurrent miscarriage and it is associated with other adverse pregnancy outcomes such as preterm delivery, intrauterine growth restriction, preeclampsia, and HELLP syndrome. Obstetric morbidity is one of the major manifestations of APS with a wide variety of clinical manifestations. This case describes a case of a severe preeclampsia in a 16-year-old primigravida at 29 weeks resulting in a caesarean delivery and subsequent finding of an ulnar artery thrombosis in postpartum period. APS was diagnosed on further investigations of her symptoms and signs.

## 1. Background

Antiphospholipid syndrome (APS) is an autoimmune disorder characterized by recurrent thrombosis and/or obstetric morbidity. Prevalence of the antiphospholipid antibodies (anti-PL) in the general population ranges between 1 and 5%. However, only a minority of these individuals develop the APS [1]. Conversely, a recent review has found that aPL are positive in approximately 13% of patients with stroke, 11% of patients with myocardial infarction, 9.5% of patients with deep vein thrombosis (DVT), and 6% of patients with pregnancy morbidity [2].

Obstetric complications are major manifestations of APS and have a serious impact on maternal and fetal morbidity. These include recurrent miscarriages and are associated with other adverse obstetric outcomes such as preterm delivery, fetal growth restriction, preeclampsia, and HELLP syndrome [3, 4].

Here we report a rare case of a severe preeclampsia associated with primary APS and ulnar artery thrombosis in a teenage pregnancy at 29 weeks of gestation. The importance of reporting is the rare association, the pattern of clinical presentation of thrombosis over a long period, and occurrence of these three conditions in the same patient.

## 2. Case Presentation

A 16-year-old, Sinhala ethnic Sri Lankan woman in her first pregnancy, was admitted with severe preeclampsia at 29 weeks of gestation. She has made her booking visit at ninth week of gestation and all the booking investigations were normal except for the platelet count which was 112,000 per liter. During her pregnancy, the lowest platelet count was 80,000 per liter at 27 weeks of gestation and no specific intervention has been done except for regular monitoring of the platelet count. She had been diagnosed with gestational hypertension at 22 weeks of gestation and prescribed labetalol and methyldopa. Other than that, she has had few erythematous, itchy macular lesions over the palm of her right hand from early in the first trimester onwards and had persisted throughout the pregnancy. She has had mild pain in her right small finger from first trimester onwards. But she had not worried about these symptoms so they had gone unnoticed. She had been apparently well until late 28 weeks of gestation and then she has developed a severe headache and worsening of bilateral lower limb oedema with frothy urine leading to hospitalization. She was diagnosed with severe preeclampsia (blood pressure of 185/115 mmHg) at 29 weeks of gestation. An emergency caesarean delivery was arranged soon after this presentation. Her baby was admitted to the premature baby unit with a birth weight of 1000 grams. She was in intensive care unit in first 24 hours after delivery and received intravenous magnesium sulphate as a prophylactic anticonvulsant.

Her pain in the right finger worsened after delivery and erythematous macular lesions have been increased in number and spreading over the dorsal aspect of the right forearm. She was not worried and lesions have gone unnoticed especially with her dark skin complexion. Her blood pressure was under control with oral nifedipine. At the eighth postpartum day, her right small finger was noted to be cold with increased pain. Discoloration of the above skin lesions was more prominent and started to appear over the palm and the ventral aspect of the forearm of the right hand too, with preserved capillary refilling time. Both radial and ulnar artery pulsations were felt. There were no similar lesions in any other part of the body. She was soon transferred to a medical ward for further management.

**Figure 1:**
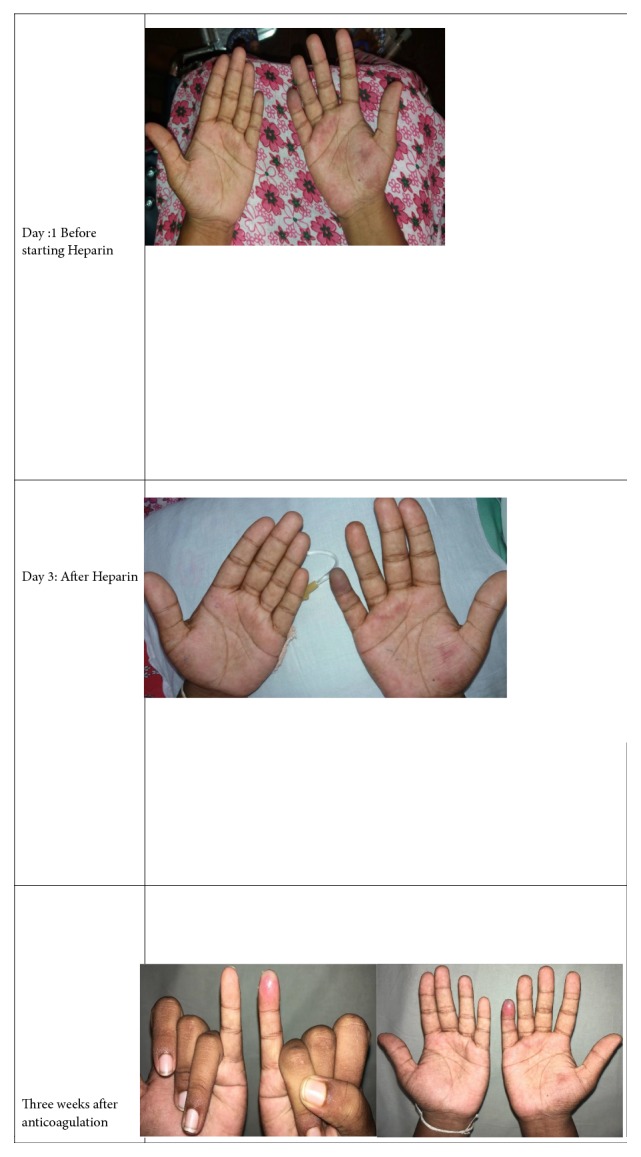
Sequential changes of affected finger with heparin/ anticoagulation treatment.

She was subjected to an urgent arterial duplex study, which revealed proximal ulnar artery thrombosis in the right side with partial occlusion to the blood flow. And soon she was started on unfractionated heparin and eventually bridged with oral anticoagulants (warfarin) in order to archive the target international normalized ratio (INR) of 2.0-3.0. With anticoagulation treatment, her symptoms and signs were markedly improved. Sequential macroscopic changes of the affected arm and fingers have been shown in Figure 1.

Routine laboratory analyses were within the normal range including subsequent platelet count, but she got positive results for direct Coombs test. Her reticulocyte count was high with normal haemoglobin concentration. Her ANA titre was strongly positive (1:320). And also anti-cardiolipin antibodies (anti-CL) and anti-*β*2 glycoprotein-I (anti-*β*2GPI) levels were also noted to be positive. However, her ds DNA and C3/C4 levels were within normal limits. Her blood pressure readings too have come back to normal level with no requirement of medications. Also proteinuria was settled. Her laboratory tests for APS were positive even after 12 weeks of initial testing. Therefore, it was diagnosed as a case of primary APS.

## 3. Discussion

APS is defined by the development of venous and/or arterial thromboses, often multiple, and pregnancy morbidity (mainly, recurrent pregnancy losses), in the presence of antiphospholipid antibodies (anti-PL), namely, lupus anticoagulant (LA), anti-CL, or anti-*β*2GPI [1, 5]. These features are linked to the presence in the blood of autoantibodies against negatively charged phospholipids or phospholipid-binding proteins [1]. The APS can be found in patients having neither clinical nor laboratory evidence of another definable condition (primary APS) or it may be associated with other diseases leading to secondary APS, mainly systemic lupus erythematosus (SLE), but occasionally with other conditions such as infections, drugs, and malignancies [1].

According to the revised classification criteria, the diagnosis of APS can be made when there is at least one positive clinical criterion along with positive laboratory tests found on at least two occasions 12 weeks apart.  Table 1 shows revised criteria for the diagnosis of APS. The guidelines published by the international society on thrombosis and haemostasis (ISTH) have mentioned the anti-*β*2GPI IgM or IgG titres exceeding 99th percentile and anti-CL levels exceeding 40 IgM and IgG phospholipid units as positive tests for APS [6]. In addition to that, there are certain other associations for the APS such as valvular heart disease, livedo reticularis, thrombocytopaenia, nephropathy, and neurological impairment which were not included in the diagnostic criteria [5]. In our patient, she has fulfilled the vascular criteria and the pregnancy criteria and the laboratory investigations also showed positive values for both antibodies in moderate to high titres in two separate occasions 12 weeks apart. Therefore, this is a case of APS presented with severe preeclampsia and arterial thrombosis in a younger age.

Preeclampsia was not considered as a major criterion for APS but its presence might favour the diagnosis of possible APS. It is reported that 18% of pregnant patients with underlying APS can present with preeclampsia [7, 8] Therefore, we suggest that, for women with severe preeclampsia or HELLP, screening for the possibility of APS would be beneficial rather than waiting until she fulfills the major criteria and it would be of much benefit in the assessment of future pregnancy outcomes as well [9]. It has been shown that severe preeclampsia is a distinct entity from nonsevere preeclampsia and is mainly associated with the presence of anti-*β*2GP1 IgG [9]. Ulnar artery thrombosis may present with a spectrum of symptoms such as Raynaud's phenomenon, digital ischemia, cyanosis, pallor, pain, and gangrene formation mainly in the 4th and the 5th digits [10]. The presentation might be variable depending on the site of thrombosis and the nature of the collateral circulation [10]. Thrombosis of the proximal arteries such as the ulnar artery is unusual without any preceding history of trauma or occupational exposure which suggests more towards underlying thrombophilic condition [10]. A recent study has revealed the knowledge between false-positive TORCH (toxoplasmosis, other: syphilis, varicella-zoster, Rubella, CMV, and herpes infections) and anti-PL opening new diagnostic opportunities, relevant for practical decisions [11]. Also, in the spectrum of vascular ischaemic occlusive disease in the APS, ulnar artery thrombosis is a rare association [12]. However, with the acute episode of thrombosis, we were unable to arrange certain other investigations to rule out any concomitant familial thrombophilic disorder screening even though the family history is not significant.

This case report adds to the literature about an uncommon case of combinations of severe preeclampsia, ulnar artery thrombosis, and APS in a teenage pregnant mother. Moreover, it also shows the value of multidisciplinary team management in these type of complex cases to yield an optimum patient care and a better long-term outcome.

## 4. Conclusion

Considering the fact that the adverse pregnancy outcomes in younger age is associated with an arterial thrombosis in an unusual site which could not be explained by the prothrombotic state of the pregnancy alone favours a strong clinical suspicion towards APS. This gives a message to clinicians to be more vigilant about the potential autoimmune origin in pregnant mothers with severe preeclampsia/HELLP presenting at a very young age.

## Figures and Tables

**Table 1:** International consensus statement on criteria for the classification of the antiphospholipid syndrome.

Clinical Criteria	Clinical and Laboratory Events
Thrombosis	Venous, arterial, or small vessel
Pregnancy morbidity	≥ 2 unexplained fetal losses(<10 weeks of gestation)
	≥ 1 unexplained fetal losses (>10 weeks of gestation)
	≥ 1 premature births of morphologically normal neonates at or before 34th week of gestation
Laboratory criteria	Anticardiolipin antibodies
	IgG or IgM present in > 40 GPL or MPL on two occasions at least 12 weeks apart
	Anti-b2GP1 of IgG/IgM > 99th percentile

Ig: immunoglobulin; *β*2GPI: *β*2 glycoprotein-1 antibodies GPL, MPL.

## References

[1] Cervera R. (2017). Antiphospholipid syndrome. *Thrombosis Research*.

[2] Andreoli L., Chighizola C. B., Banzato A., Pons-Estel G. J., De Jesus G. R., Erkan D. (2013). Estimated frequency of antiphospholipid antibodies in patients with pregnancy morbidity, stroke, myocardial infarction, and deep vein thrombosis. *Arthritis Care & Research*.

[3] Branch W. (2011). Report of the Obstetric APS Task Force: 13th International Congress on Antiphospholipid Antibodies, 13th April 2010. *Lupus*.

[4] Galarza-Maldonado C., Kourilovitch M. R., Pérez-Fernández O. M. (2012). Obstetric antiphospholipid syndrome. *Autoimmunity Reviews*.

[5] Miyakis S., Lockshin M. D., Atsumi T. (2006). International consensus statement on an update of the classification criteria for definite antiphospholipid syndrome (APS). *Journal of Thrombosis and Haemostasis*.

[6] Lim W. (2013). Antiphospholipid syndrome. *International Journal of Hematology*.

[7] Lima F., Khamashta M. A., Buchanan N. M., Kerslake S., Hunt B. J., Hughes G. R. (1996). A study of sixty pregnancies in patients with the antiphospholipid syndrome. *Clinical and Experimental Rheumatology*.

[8] Branch D. W., Silver R. M., Blackwell J. L., Reading J. C., Scott J. R. (1992). Outcome of treated pregnancies in women with antiphospholipid syndrome: an update of the Utah experience. *Obstetrics & Gynecology*.

[9] Marchetti T., de Moerloose P., Gris J. C. (2016). Antiphospholipid antibodies and the risk of severe and non-severe pre-eclampsia: The NOHA case-control study. *Journal of Thrombosis and Haemostasis*.

[10] Carpentier P. H., Biro C., Jiguet M., Maricq H. R. (2009). Prevalence, risk factors, and clinical correlates of ulnar artery occlusion in the general population. *Journal of Vascular Surgery*.

[11] De Carolis S., Tabacco S., Rizzo F. (2018). Association between false-positive TORCH and antiphospholipid antibodies in healthy pregnant women. *Lupus*.

[12] Atanassova P. A. (2007). Antiphospholipid syndrome and vascular ischemic (occlusive) diseases: An overview. *Yonsei Medical Journal*.

